# Marcus Gunn Syndrome in Primary Care: A Case Report

**DOI:** 10.7759/cureus.43738

**Published:** 2023-08-19

**Authors:** Carlota Barreira, Rita Barreira

**Affiliations:** 1 General Practice, USF Ajuda, ACES Lisboa Ocidental e Oeiras, Lisbon, PRT; 2 Pediatrics, Hospital São Francisco Xavier, Lisbon, PRT

**Keywords:** eyelid ptosis, congenital palpebral ptosis, jaw winking, ptosis, marcus gunn syndrome

## Abstract

Marcus Gunn syndrome (MGS) is a rare craniofacial condition characterized by abnormal eyelid movements synchronized with jaw muscle activity. This case report describes a one-month-old girl with right eyelid ptosis and involuntary movements of the right eyelid during sucking. The diagnosis of MGS was made based on clinical observations. The etiology of MGS is not well-defined, and long-term follow-up is necessary to assess the progression of the condition. Early referral to ophthalmologists and neurologists/pediatricians is important to evaluate concomitant conditions and prevent secondary complications. Primary care physicians, who maintain continuous contact with patients, play a crucial role in detecting initial symptoms, initiating appropriate investigations, and coordinating multidisciplinary care. By raising awareness among primary care physicians about the signs, symptoms, and referral pathways for MGS, this case report aims to improve the recognition and management of this rare condition in primary care settings. Emphasizing the role of family doctors in the early identification and referral of MGS can lead to better outcomes for affected patients.

## Introduction

Marcus Gunn syndrome (MGS), which is also called the Marcus Gunn phenomenon or Marcus Gunn jaw-winking trigemino-oculomotor synkinesis, was first described by Robert Marcus Gunn in 1883 in a female with unilateral blepharoptosis with associated upper eyelid contraction on chewing movements. It is characterized by the presence of abnormal eyelid movements during sucking or chewing, which are synchronized with jaw muscle activity and predominantly occur unilaterally [1,2]. Males and females are equally affected [3], with recent reports suggesting that this condition presents in approximately 5% of neonates with congenital ptosis [3]. It is a congenital condition, albeit acquired forms may occur due to complications from surgery, trauma, infections, or pontine tumors [4].

Although the underlying etiology of MGS is still poorly understood, throughout the literature, two main theories emerge as possible explanations for MGS. The first proposes the concept of an aberrant connection in which a structural abnormality in the brainstem causes neural misdirection of trigeminal motor axons to the levator palpebrae superioris (LPS) muscle [5,6]. The second hypothesis is called the “release hypothesis” and postulates that a primitive masticatory oculomotor reflexive circuit, which is normally suppressed in humans, is preserved, and it can be released becoming active because of intrauterine trauma or some unknown causes, resulting in the synkinetic lid and jaw movement [7].

The diagnosis of MGS is primarily clinical [8], and there is no specific treatment. Some patients may learn to control synkinesis and thus may show improvement in the condition. Surgery is usually considered when the jaw-winking or blepharoptosis represents a significant functional or cosmetic problem and in cases associated with amblyopia or vertical strabismus [9]. Despite being a rare condition, it is crucial for healthcare professionals to be familiar with it to allow an early diagnosis and appropriate management.

## Case presentation

A one-month-old girl presented for the first time to the primary healthcare center. Since birth, parents noticed involuntary movements of the right eyelid associated with sucking during breastfeeding. The parents are non-consanguineous, and the pregnancy was adequately monitored with a ventouse delivery at 39 weeks without complications. There is no history of birth trauma and no relevant family history. The patient is exclusively breastfed, supplemented with vitamin D, with adequate psychomotor development and growth.

On physical examination, she displayed rhythmic elevation of the right eyelid occurring consistently when sucking on a pacifier, with otherwise-normal findings on physical and neurologic examinations (Video 1). The pediatric clinic confirmed the diagnosis of MGS based on the findings, and the patient was subsequently referred for observation by ophthalmology to rule out strabismus or amblyopia. Ophthalmic examination was normal, including external, anterior, and posterior segment structures, extraocular motility in both eyes, and the cover test. The pupillary examination was also normal. The follow-up is performed by ophthalmology every year.

**Video 1:** Elevation of the right eyelid due to suction

## Discussion

MGS is a rare disease varying in severity, with parents in most cases noticing in the first few weeks after birth an eyelid retraction during the child's feeding, as was the case in this patient. Some authors suggest that ptosis does improve over time, but there is so far no evidence to support this claim, emphasizing the need for a long-term follow-up of these patients (from six months to once a year) [10]. It is believed that over time, affected individuals are able to recognize which movements are responsible for the synkinesis and learn how to control or avoid them, thereby minimizing its effects.

It is important to highlight that patients with a probable diagnosis of MGS should be referred early to ophthalmology and pediatric neurology consultation due to the possibility of associated conditions and secondary complications such as strabismus and anisometropia, present in 50%-60% and 5%-25% of cases, respectively [3]. Regarding differential diagnosis, other synkinetic alterations that present with eyelid ptosis should be considered such as inverse Marcus Gunn phenomenon and Marin-Amat syndrome. Both involve ptosis that becomes more evident with jaw movement. The former is a congenital condition characterized by an inhibition of the LPS muscle [10] and the latter is an acquired synkinesis occurring most frequently after Bell's palsy [11].

## Conclusions

MGS is a rare and benign entity with a favorable prognosis that remains relatively unknown. The objective of this clinical case is to raise awareness among primary care physicians about the importance of establishing a close relationship with both parents and patients in the early years of life to enable an early diagnosis and subsequent treatment whenever appropriate. A multidisciplinary diagnostic approach is mandatory, and primary care physicians, who maintain privileged contact with the child since birth, play an essential role. Further research is also needed to better understand the underlying mechanisms of this syndrome, enabling the development of more specific therapeutic approaches.
